# Semi-Supervised Anomaly Detection in Video-Surveillance Scenes in the Wild

**DOI:** 10.3390/s21123993

**Published:** 2021-06-09

**Authors:** Mohammad Ibrahim Sarker, Cristina Losada-Gutiérrez, Marta Marrón-Romera, David Fuentes-Jiménez, Sara Luengo-Sánchez

**Affiliations:** Department of Electronics, Politechnics School, Campus Universitario S/N, University of Alcalá, Alcalá de Henares, 28801 Madrid, Spain; ibrahim.sarker@uah.es (M.I.S.); marta.marron@uah.es (M.M.-R.); d.fuentes@edu.uah.es (D.F.-J.); sara.luengo@edu.uah.es (S.L.-S.)

**Keywords:** anomaly detection, RGB, CNN, multiple instance learning, video-surveillance

## Abstract

Surveillance cameras are being installed in many primary daily living places to maintain public safety. In this video-surveillance context, anomalies occur only for a very short time, and very occasionally. Hence, manual monitoring of such anomalies may be exhaustive and monotonous, resulting in a decrease in reliability and speed in emergency situations due to monitor tiredness. Within this framework, the importance of automatic detection of anomalies is clear, and, therefore, an important amount of research works have been made lately in this topic. According to these earlier studies, supervised approaches perform better than unsupervised ones. However, supervised approaches demand manual annotation, making dependent the system reliability of the different situations used in the training (something difficult to set in anomaly context). In this work, it is proposed an approach for anomaly detection in video-surveillance scenes based on a weakly supervised learning algorithm. Spatio-temporal features are extracted from each surveillance video using a temporal convolutional 3D neural network (T-C3D). Then, a novel ranking loss function increases the distance between the classification scores of anomalous and normal videos, reducing the number of false negatives. The proposal has been evaluated and compared against state-of-art approaches, obtaining competitive performance without fine-tuning, which also validates its generalization capability. In this paper, the proposal design and reliability is presented and analyzed, as well as the aforementioned quantitative and qualitative evaluation in-the-wild scenarios, demonstrating its high sensitivity in anomaly detection in all of them.

## 1. Introduction

In recent years, surveillance cameras have gained high popularity for taking care of public safety against the increased security threats in various forms, such as robbery, accidents, or illegal and anti-social activities. Nowadays, these cameras are commonly installed in public places such as banks, shopping markets, railway and bus stations, crowded streets or heavy traffic areas, which are liable to security threats, in order to guarantee public safety.

Traditionally, it has been an operator who is responsible for reviewing the video-surveillance images, and determines the existence of abnormal or dangerous events. However, manual detection of these anomalies from all the sequences captured by a camera is labor and time intensive, and it requires a dedicated person to be employed for this purpose. In addition, the efficiency of detection relies on the potential of the individual. The task also becomes monotonous and boring as the occurrence of abnormal events is very minimal when compared to that of the normal events. This drawback may lead to the under-utilization of surveillance cameras. Hence, automation of this anomaly detection task finds its applicability in several industrial contexts such as security guards, traffic security, or crime prevention.

In the context of automating anomaly identification from surveillance videos, computer vision algorithms can be employed to sense and notify the abnormal events along with the time frame within which these have occurred. As a first step, algorithms for anomaly detection should be able to identify whether a specific video displays anomaly events or not. If an anomaly is detected, it can be further processed to categorise it among the many kinds of anomalies that may appear in a video-surveillance scenario: robbery, accidents, burglary, etc.

Several methods have already been developed by the scientific community in order to solve the automatic anomaly detection tasks in video-surveillance situations. The very first ones were based on simple and specific anomaly detectors, such as accident, violence, or illegal activities detectors based on image features. Violent flow descriptors [1] and heuristic approaches [2] have also been employed to distinguish violent from non-violent videos. However, these specialized anomaly detectors are just useful to identify events that fall under a specific anomaly type. Thus, their applicability is limited.

In real world environments, anomalous events are complex, miscellaneous, and most importantly, unexpected. Hence, it is difficult to list all of them beforehand, and, thus, to design a detector reliable in all scenarios. Therefore, it is preferable that anomaly detection algorithms can identify these anomalies without prior knowledge about them, that is, in an unsupervised or weakly supervised way.

The earlier attempts towards anomaly detection can be categorized into supervised and unsupervised approaches. Unsupervised approaches build a training model based on normal events and then anomalies are detected based on the reconstruction error during the testing phase.

However, this approach results in a high number of false positives as the normal events itself will be radically changing over time and the latter normal events can be detected as anomalous. Alternatively, supervised methods can be used. In this case, both the normal and the anomalous videos are taken into consideration for training, appearing the already mentioned problems of obtaining annotations for every video segment and type. To overcome this global issue, weakly supervised learning techniques are explored, since these methods combine the benefits from supervised approaches, without needing an exhaustive labeling.

In this context, this work presents a proposal for anomaly detection based on multiple instance learning (MIL), a weakly supervised learning approach. The proposed work, thus, aims at contributing to anomaly detection in surveillance videos with improved performance, that besides demonstrates a better capability of generalization. In this regard, the primary contributions of this work include:

(1) Employing temporal convolution 3D network (T-C3D) for feature extraction, trained with the noise-free large scale Kinetic dataset;
(2) Formulating a modified ranking loss function that minimizes the miss rate noticeably, improving the anomaly detection performance;
(3) Describing a global proposal, based on MIL, that demonstrates a high generalization capability for anomaly detection in unseen real scenarios in the Wild, understanding “in the Wild” as in non-prepared daily scenarios, instead of in lab-based ones [3,4].

The rest of the paper is structured as follows. Section 2 presents the related work pertaining to anomaly detection. Section 3 describes the proposed methodology. Section 4 discusses the dataset and reports the main results and Section 5 concludes the paper and presents different future works.

## 2. Related Works

Several works have been previously proposed towards anomaly detection in surveillance videos, most of them, using different machine learning and deep learning [5,6,7].

The different proposals can be categorized as supervised, unsupervised, and weakly supervised approaches. Though the first ones have demonstrated to be really useful and reliable in some scene understanding tasks (such as object detection or action recognition), this is not the same in the anomaly detection problem, given the difficulty of modeling all versions of a given anomalous element in a specific context but with open conditions, as it is the case in video-surveillance. In this context, unsupervised proposals have thus given very promising results in recent years’ state-of-the-art.

Unsupervised methods that require minimal or no supervision have been employed within different approaches by the scientific community to detect anomalies in videos. These algorithms build training models from normal events while anomalies are detected based on reconstruction error during the testing phase. They can be grouped as reconstruction, spatio-temporal prediction, and generative models.

The first approaches were focused on detecting anomalous trajectories in video-surveillance scenes. A very interesting framework, based on pixel level anomaly probability density functions, is proposed in [8]. Globally understanding, the approach analyzes objects size in relation to height and width and their movement patterns, defined by their tracks: destination location and transition time. This work opens a spatio-temporal pattern and local features based approach, extensively exploited within the deep learning context in further and more modern proposals, as the one presented in this paper. A similar approach based on particle o probabilistic hypothesis trajectories is presented in [9]. Again, this proposal tackles the problem in the dual spatio-temporal approach, however, tracking remains complex and unreliable, and hence most of posterior methodologies adopt some other unsupervised approaches to detect anomalies.

Lastly, a novel method is presented in [10] for abnormal activity region detection from video-frames by utilizing prediction and measurement method. Particle weight and particle estimation for variable regions are created using shifting cell structures in the video frames. Location and motion features are then extracted using shifting regions for efficient estimation of abnormal videos and frames.

Besides, in the pattern recognition area, a histogram of orientation magnitude and entropy with fast accelerated test (also called HOME FAST) spatio-temporal descriptor, for feature creation to estimate the anomaly in video frames, is defined in [11]. The created filter is utilized in smart systems for robust anomaly prediction, by means of a deep learning model trained with video frames to learn normal activity patterns. This model efficiently predicts the local and global abnormal activity patterns, showing a competitive performance while dealing with local and global anomaly environments.

Finally, in the context of unsupervised reconstruction-based models, also sparse combination learning techniques have been adopted in [12] to detect anomalies in surveillance contexts.

More recent works use encoder-decoder structures, or auto-encoders, for anomaly detection. In [13], it is presented a hybrid combination of long short-term memory (LSTM) encoder-decoders and auto-encoders used to get spatio-temporal information in video segments, with which the presence or absence of anomaly is detected is presented in [13] as an accurate approach. Additionally, authors of [14] propose to use a similar structure of auto-encoder and LSTM for detecting video anomalies in an unsupervised way.

There are some other interesting works that use auto-encoders for spatio-temporal prediction. In [15], firstly, spatio-temporal local features from an anomaly video-surveillance dataset have been utilized to train a fully connected auto-encoder. Then, both these local features, as well as neural network based classifiers, have been used to train a fully convolution feed forward auto-encoder. Having thus reconstructed the model, anomaly detection is performed by comparing the obtained error between testing videos and their modeled-output.

Again within this context of spatio-temporal prediction, a deep neural network (DNN) based spatio-temporal auto-encoder has been designed to detect anomalies in [16]. In this different proposal, the encoder perceives a video representation to obtains from it both spatial and temporal features separately. Moreover, a weight decreasing prediction loss function has been incorporated to improve the learning rate of features related to motion. Similarly, in [17], the authors propose the use of a dictionary based learning method, where a latent space of variational auto-encoder is used.

Finally, the authors of [18] propose a three-stage unsupervised anomaly detection method that, instead of auto-encoders, uses a random projection in the first stage extracted features to improve performance, followed by a refinement network and inference ensemble stages. In the same direction, generative adversarial networks (GAN) have also been trained to learn normal events [19], so during testing, discriminators of normal events are utilized to detect abnormal events. The same idea is present in [20], where a method to predict future frames based on spatial intensity, gradient, and motion characteristics in order to identify anomalies if the predicted frame differs from the actual is described. In this order, sparse coding-based methods (reconstruction based) achieve decent anomaly detection accuracy. These methods are built on the basis that the initial few frames of the video characterize normal events and, hence, can be utilized to construct a dictionary for normality. Then, anomalous events are identified on the fact that they cannot be accurately reconstructed from the normal event database.

The above-stated unsupervised approaches perform well if normal events are similar all the time and the abnormal ones differ from them noticeably. However, generally, in real applications normal events themselves may change drastically and hence these approaches result in high false-positive rates. Alternatively, supervised approaches [21,22] have also been widely used towards anomaly detection, due to the promising results exhibited by deep learning approaches in different computer vision applications.

There are only few works that address anomaly detection using approaches based on supervised learning, mainly due to the lack of large annotated datasets. In this context, the proposal described in [23] uses a graph convolutional neural network to correct noisy labels for anomaly detection. The noise correction is carried out based upon a feature similarity and temporal consistency. Then, anomaly detection is performed in a fully supervised way, with high accuracy, by classifying the actions in the scene. Additionally, the proposal in [24] is based on a supervised approach. The authors propose to use a light-weight CNN followed by a residual attention-based LSTM for anomaly detection. The light-weight CNN reduces time complexity allowing to obtain competitive accuracy.

As it has been stated before, there is a lack of large annotated datasets for anomaly detection, thus, most of the supervised proposals are based on action recognition. This is because supervised learning algorithms require a large amount of annotated samples for both normal and anomalous events. Furthermore, all the anomalous events to be detected must be included in the training stage. Besides, supervised approaches only works in controlled conditions in which there are available annotated samples for both, normal and abnormal events that must be detected.

Taking into account the inconveniences that both supervised and also unsupervised approaches present in video-surveillance anomaly detection, semi-supervised techniques appear to be good solutions for this problem. These approaches usually provide better accuracy that unsupervised ones, and require the annotation in the video level, but not for every video segment or frame. Thus, it is avoided the need for thorough labeling, which, on one hand, is not always possible and on the other hand constraints too much the normality, leading to a high false positive rate in anomaly detection. Thus, weakly supervised learning builds training models based on the weakly labelled data but predicts the label for every frame during testing.

In this context of weakly supervised approaches, multiple instance learning (MIL, firstly cited in [25]) technique has been widely used. The base of the technique is to build the training model from labelled bags (comprising of multiple instances) rather than labelled individual instances. A bag is annotated as negative if all the instances in it are negative while it is marked positive if at least one instance in it is positive. Then, the learned model must be able to predict labels for individual instances.

There are several proposals based on MIL for anomaly detection in video sequences. The approach described in [26] use inflated 3D pre-trained from Kinetics dataset to extract features. These features are passed through an anomaly regression network (AR-Net) which requires video level label for anomaly detection. Similarly, the proposal in [27] uses a graph-based MIL model whose output is used to improve a dictionary. Anomalous events are then detected based on the sparse reconstruction cost (SRC).

Another proposal based on MIL is the one in [28]. In this work, outer bag loss, associated with the difference in score between abnormal and normal bags, is formulated to push apart the anomalous and normal bags from each other. The above-stated work has been extended to include inner bag loss in [29] as intra-difference between scores within a bag should be as low as possible.

Previously described works, based on MIL [28,29], can detect some anomalous sequences, but in some cases, the anomaly scores are low, mainly due to the high variability of the anomalous events in video-surveillance. Therefore, some anomalous video-segments are detected as normal ones, appearing a high number of false negatives.

As it has been explained in this section, there exists several proposals for anomaly detection in the literature, based on unsupervised, semi-supervised, and fully-supervised deep learning techniques. To avoid the drawbacks of the unsupervised an fully-supervised methods, this paper presents a novel approach based on MIL. Moreover, to reduce the number of false negatives that appear in the described MIL proposals, there is introduced a new ranking loss function that is able to reduce the variability for anomalous sequences and increases the difference between the scores for normal and abnormal sequences, improving the accuracy. The proposed approach is described in detail below.

## 3. Methodology

### 3.1. Proposed Architecture and Training Procedure

**Figure 1:**
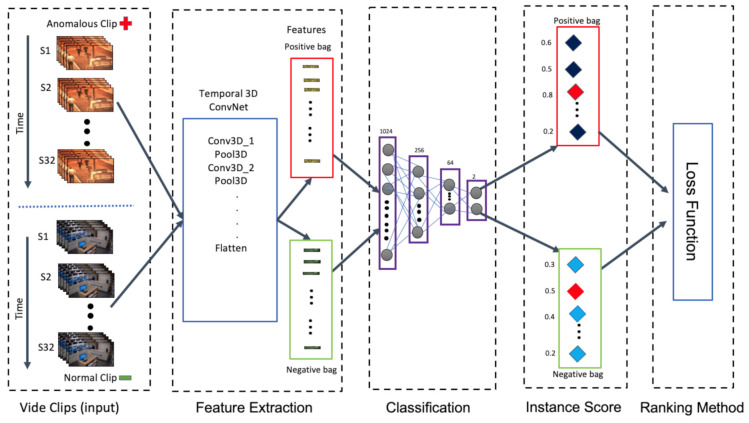
Proposed architecture for anomaly detection in surveillance videos.

The proposed methodology towards anomaly detection in surveillance videos using MIL is presented in detail in this section. In order to overcome the challenge in anomaly detection, we proposed an architecture as shown in Figure 1.

Because of the memory bottleneck, the whole video cannot be fed to the model at once. So every video in the training and testing sets is split into 32 non-overlapping video segments (which is set after several experiments, and a standard in the state of the art), then the spatio-temporal features are extracted for each video segment using a T-C3D [30] as a feature extractor. We have also observed that using 64 non-overlapping video segments performs better on longer videos.

The proposed T-C3D divides the video into multiple frames that are them utilized to make a clip. The created clips using video frames are used as an input to the 3D convolutional network. The robust 3D features from videos are extracted using extended 2D temporal dimensions, being the weights of 3D-CNN the same on the entire video clip. Features extracted from different videos are fused by using the aggregation function to determine the class score for the prediction of video clip. Thus, the proposed T-C3D computes the loss value using the loss function and perform predictions on videos. The implanted T-C3D model deals with all the parameters, features, and predictions at the video instead of single frames. Video clips of all categories are created, and the regression function combines the clips of the same categories into a specific video, from what the network is able to estimate the probability of each activity. In our proposed work the output layer is the last fully connected layer. The implementation of back-propagation allows differentiating the temporal encoding method. The use of logarithmic loss function allows to compare segmentation process to achieve the final segmented region will be expressed as:

(1) $$M\left(y,H\right)=\sum_{i=1}^{N}y_{i}\left(H_{i}-\log\sum_{j=1}^{N}\exp H_{j}\right)$$

The number of classes is represented by *N* and $y_{i}$ denotes the ground-truth label of prediction class *i*. *H* is the logarithmic consensus loss function of segmented regions. Aggregation and regression derivable functions enable the multiple clips to update the network weights *W* using the traditional back-propagation algorithm. The back-propagation gradient weights and loss value *L* can be defined by Equation (2):

(2) $$\frac{\partial L(y,H)}{\partial W}=\frac{\partial L}{\partial W}\sum_{s=1}^{S}\frac{\partial Q}{\partial F\left(C_{a}\right)}\frac{\partial F\left(C_{a}\right)}{\partial W}$$

T-C3D adopted video clips are denoted by *W*. In the above equation *H* is derived from clip prediction using the optimization of weights by segmentation concurrence. *Q* describes the aggregation function which contains the fusion of multiple clips of videos. The proposed T-C3D is utilized for feature extraction from a noise-free abnormality detection dataset.

The T-C3D learns video actions through a hierarchical multi-granularity method, that gives spatial information from a single image, and temporal information when provided with a sequence of images. Thus, it encapsulates the temporal dynamics of the entire video through a temporal encoding approach, enhancing the model real-time inference speed. T-C3D considers the whole video level features while calculating the loss function. This makes the model enable to learn hard examples more efficiently. Instead of feeding the whole video at once T-C3D feed batch of segments and use an aggregation function to gain video-level features.

Some previous works that incorporate long-term temporal convolution (LTC) [31] and 3D CNNs show that 3D models pre-trained with Sports-1M [31] demonstrate better performance than those trained from scratch. Sports-1M dataset is composed of more than one million videos, but it includes some noise owing to a lack of manual annotation. Recently, ref. [32], introduce the Kinetics dataset that comprises 400 human action categories with at least 400 video samples for each one. This dataset is, thus, large-scale and free from noise. Because of that, in this proposal, T-C3D is pre-trained using the Kinetics dataset, instead of Sports-1M, with the view to improve the accuracy of the proposed anomaly detector.

The generated feature vector from T-C3D is used to label the video segment as positive (presence of anomaly) or negative (absence of anomaly). This feature vector is further processed at the classifier network (deep MIL network) which is included at the end of T-C3D feature extractor. It consists of a 3D average pooling layer, followed by four fully connected layers, also including batch normalization. A dropout of 60% is incorporated in the ReLU activation function.

In order to train the deep MIL framework, instances are grouped into bags as it necessitates the annotations at only video level rather than at video segment level. The bag of features which contains at least one positive video segment (with anomaly) is labelled as a positive bag $B_{a}$, while the bag where all the video segments correspond to negative ones is considered to be a negative bag $B_{n}$. The algorithm learns and builds a model from the labelled bags. The objective function is applied based on the aggregation of scores of individuals in the bags.

In this context, the anomaly detection is viewed as a regression problem. Therefore, classification scores to $B_{a}$ and $B_{n}$ are predicted for each video segment.

The obtained scores are provided as input to the the loss function f(v), so it is desirable that video segments with anomalies $f(v_{a})$ are characterized by higher scores than those without anomalies $f(v_{n})$, (Equation (3)), where $v_{n}$ and $v_{a}$ refer to normal and anomalous video segments, respectively.

(3) $$f\left(v_{a}\right)>f\left(v_{n}\right),$$

Nevertheless, ground truth annotations are given only at video-level rather than at video segment-level, thus minimizing labeling time and complexity. Because of that, the relationship in Equation (3) cannot be used for this work. Besides, it is proposed a new multiple instance ranking loss that is explained in Section 3.2.

In addition, instead of using an adaptive gradient algorithm (AdaGrad) [33], it is employed Adadelta [34] as the optimizer, since an empirical analysis performed in this work concludes that AdaGrad is too aggressive in decreasing learning rates. Adam algorithm was also tested, but it demonstrated a large momentum minimization error.

To pre-train TC3D with Kinetics 400 dataset took 13 weeks with our system. During training of the UCF-Crime dataset we run a total of 60k iterations and decrease the learning rate by half at 20k, 40k, and stop at 60k with a total of 3.5 weeks of training time.

### 3.2. Proposed Ranking Loss Function

Our proposed technique is inspired by [28] using MIL. The concept considers the input videos as binary instance. The negative instances are the videos with normal activity whereas positive instance videos contains abnormal activity. Abnormal activity is a small segment of the whole video and all the video contains these segments of anomalies. The baseline method computes the loss function as follows:

(4) $$\max_{i\in B_{a}}f\left(v_{a}^{i}\right)+\max_{i\in B_{n}}f\left(v_{n}^{i}\right)$$

According to Equation (4), a bag is assigned the highest score among all the video segments in that bag. The logic of this ranking objective is that the video segment with the maximum score in a positive bag (marked with red in Figure 1) should rank higher than that of the one with the maximum score in the negative bag (marked with green in Figure 1). The baseline loss function for normal and abnormal instance classification as follows:

(5) $$\begin{array}{c} L\left(B_{a},B_{n}\right)=\max\left(0,1-\max_{i\in B_{a}}f\left(v_{a}^{i}\right)+\max_{i\in B_{n}}f\left(v_{n}^{i}\right)\right) \end{array}$$

We derived (6) from baseline (4) and (5).

(6) $$\begin{array}{c} L\left(B_{a},B_{n}\right)=\max\left(0,1-\max_{i\in B_{a}}f\left(v_{a}^{i}\right)+\max_{i\in B_{n}}f\left(v_{n}^{i}\right)\right)+\\ \lambda_{1}\sum_{i}^{n-1}{\left(f\left(v_{a}^{i}\right)-f\left(v_{a}^{i+1}\right)\right)}^{2}+\lambda_{2}\sum_{i}^{n}f\left(v_{a}^{i}\right) \end{array}$$

where normal and abnormal activity segments recognition is denoted by $f\left(v_{a}^{i}\right)$ and $f\left(v_{n}^{i}\right)$, respectively. The term max elaborated all activity segments of videos in the instances. Sparsity is denoted by $\sum_{i}^{n}f\left(v_{a}^{i}\right)$ and smoothness is denoted by $\sum_{i}^{n-1}{\left(f\left(v_{a}^{i}\right)-f\left(v_{a}^{i+1}\right)\right)}^{2}$ in the mathematical modeling.

The ranking loss function in Equation (3) allows classifying the video-segments in normal or anomalous, however, due to the high variability of the anomalous events, in some cases the anomaly scores are low and similar to the normal ones. Therefore, some anomalous video-segments are detected as normal ones, appearing a high number of false negatives. Our proposed loss function depicts that the baseline method has large distributions for normal; and abnormal activity which makes the method more complex for accurate prediction of activity. The data driven baseline method with high distribution scores near the ground truth distributions is efficient in classical method of classification. The proposed method has the log base loss function to achieve better distribution is expressed as shown in Equation (7).

(7) $$L\left(B_{a},B_{n}\right)=-\sum_{i=1}^{s}Q\left(f\left(v_{a}^{i}\right)\right)\log\left(Q\left(f\left(v_{n}^{i}\right)\right)\right)$$

where, the batch of all videos is denoted by $L\left(B_{a},B_{n}\right)$ and distribution density of $f\left(v_{a}^{i}\right)$ is predicted by using the $Q\left(f\left(v_{n}^{i}\right)\right)$ function. Logarithmic function reduces the variability for anomalous sequences and increase the difference between the scores for normal and abnormal sequences, thus reducing the number of false negatives. The proposed ranking loss function derived from [28] is as follows:

(8) $$\begin{array}{c} L\left(B_{a},B_{n}\right)=max\left(0,1-\max_{i\in B_{a}}f\left(v_{a}^{i}\right)+\max_{i\in B_{n}}f\left(v_{n}^{i}\right)\right)\\ +\max\left(0,\max_{i\in B_{n}}f\left(v_{n}^{i}\right)-\log_{2}\left(\max_{i\in B_{a}}f\left(v_{a}^{i}\right)\right)\right) \end{array}$$

This loss function aims at diminishing the miss rate, that is, anomalous videos will not be missed and declared as normal. This is a valuable decision as in applications related to anomaly detection, since miss rate is a significant parameter and it should be kept as low as possible. However, it does not consider specific temporal structures of anomalies. As anomalies tends to happen for short times, the temporal structure of videos in bags of anomalous segments tend to be sparse. Moreover, the score should characterize smoothness as it should be continuous. Hence, a regularization term that incorporates the difference in anomaly scores between successive video segments as formulated in [35] is incorporated to the proposed function. Smoothness and sparsity constraint is included in the proposed ranking function, finally used in this work, is reformulated as follows:

(9) $$\begin{array}{c} L\left(B_{a},B_{n}\right)=\max\left(0,1-\max_{i\in B_{a}}f\left(v_{a}^{i}\right)+\max_{i\in B_{n}}f\left(v_{n}^{i}\right)\right)\\ +\max\left(0,\max_{i\in B_{n}}f\left(v_{n}^{i}\right)-\log_{2}\left(\max_{i\in B_{a}}f\left(v_{a}^{i}\right)\right)\right)\\ +\lambda_{1}\sum_{i}^{n-1}{\left(f\left(v_{a}^{i}\right)-f\left(V_{a}^{i+1}\right)\right)}^{2}+\lambda_{2}\sum_{i}^{n}f\left(v_{a}^{i}\right) \end{array}$$

The proposed final logarithmic loss function along with T-C3D features enhances the system robustness. Loss function helps the T-C3D to derive robust features from video frames by removing redundancy. The feature extraction is performed at a continuous frame level using a MIL hierarchical architecture for a better understanding of the whole video. Besides, the use of loss function decreases the information loss and miss rate at the training level which enables the network to learn the most relevant features. In the proposed technique loss function and fine-tuned T-C3D are, thus, the main factors impacting the system performance.

## 4. Experimental Results and Discussion

### 4.1. Experimental Setup

The proposed method of anomaly detection algorithm which is presented in this paper is analyzed both quantitatively and qualitatively. Our proposed method has been validated on three different datasets: the widely used UCF-Crime [28], GBA [36] and the Web dataset [37]. Firstly, UCF-Crime dataset is described, then the details of GBA and the Web datasets are also provided. It is to be mentioned that the UCF-Crime dataset is used to train and test the proposal, whereas GBA and The Web dataset are just used to test the final solution in an unrestricted and uncontrolled standard scenario, thus validating the system design in a real application, where no fine-tuning is performed.

The UCF-Crime dataset [28] includes both training and testing samples, in indoor and outdoor scenarios. The training ones are labeled at the video level while the testing video samples are annotated both at video segment and video levels. There are 1610 videos available for training, out of which 800 do not contain any anomalies and 810 contain anomaly video segments. There are 290 videos for testing the effectiveness of the proposed methodologies. Out of the 290 videos, 140 videos characterize anomalies while the remaining 150 videos do not exhibit any abnormality. The dataset exploits 13 different kinds of anomalies, including abuse, arrest, arson, assault, burglary, explosion, fighting, robbery, road accidents, shooting, stealing, shoplifting, and vandalism. Table 1 shows a summary of the number of videos in the UCF-Crime dataset and its distribution for training and testing.

GBA dataset [36] comprises of around 3000 indoor video sequences characterized by a resolution of 1280×720 pixels. It includes various individuals performing 4 different actions: walk, run, sit down, and fall down. All these sequences have been recorded at the Polytechnics School of the University of Alcalá, with a GoPro HERO4 camera during different days. Hence the dataset also includes significant variations in lighting. In the GBA dataset, labels are provided for each image. For each person, both his position and performed actions are annotated.

**Figure 2:**
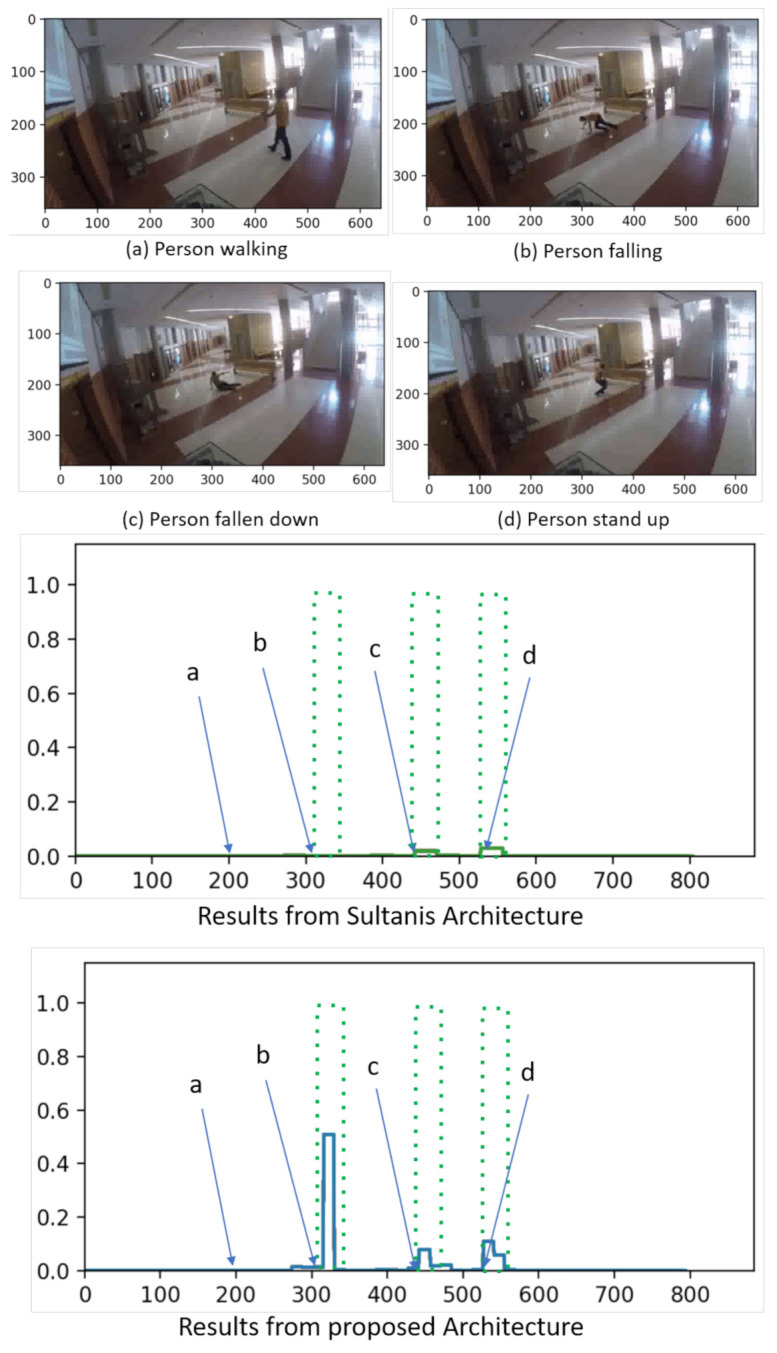
Qualitative visual results and comparison in scenes from GBA.

Table 2 presents a summary of the GBA dataset characteristics. For testing purposes, videos corresponding to people falling down are considered as anomalous, whereas the other actions correspond to normal behavior. Some sample images corresponding to the anomalous action ’Fall down’ in GBA dataset are shown in Figure 2.

The proposal in this work is also tested on the dataset of normal and crowd scenes called The Web [37]. It contains high quality crowd and normal videos in different scenes which are collected from sites such as Getty Images and ThoughtEquity.com. This dataset contains 12 normal crowd videos (pedestrian walking, marathon running) and 8 abnormal videos (escape panics, protesters clashing, crowd fighting, etc).

For the authors knowledge, UCF-Crime and The Web datasets are the most well known and referenced by the scientific community for this context [28,29], due to its diversity and size, being this the reason for its selection for training and testing the proposal here included. GBA is also included, as it comprises indoor anomalous scenes from standard in the wild environments [36], of high interest in security applications.

### 4.2. Experimental Results and Performance Comparison

The proposed method has been implemented using Pytorch. All the experiments have been performed on a PC including an intel core i7 8th generation equipped with 32 GB of RAM and 11 GB of NVidia GTX1080Ti graphic card.

In this section, there are presented the main result obtaining in different datasets, as well as a comparison to other state-of-the-art approaches. Thus, the proposed method has been tested on three challenging anomaly detection datasets: GBA, UCF-Crime, and Web dataset. Different performance evaluation measures (PEM) are calculated to validate the proposed method’s performance. The PEMs include f1-score, precision, recall [38], area under the curve (AUC) and receiver operating characteristic (ROC).

Most of the works in the literature that address anomaly detection use AUC and ROC curves for presenting their results. In this work, there are use both of them for comparison with other established works. AUC represents the area under the ROC curve, and it does not depend on the threshold, but provides a probability distribution of data which allows the positive class to rank above the negative one.

Table 3 provides a performance comparison of the proposed model with baseline method proposed in [12,15,28,29], displaying as classification quality value the AUC for each proposal tested in UCF-Crime dataset, as well as information about the kind of approach (supervised, unsupervised, or weakly supervised). In this table, it can be easily concluded the contribution of weakly supervised classifiers in anomaly detection, as explained in the contribution, and how the proposal here presented improves the results in this task from the state of the art.

**Figure 3:**
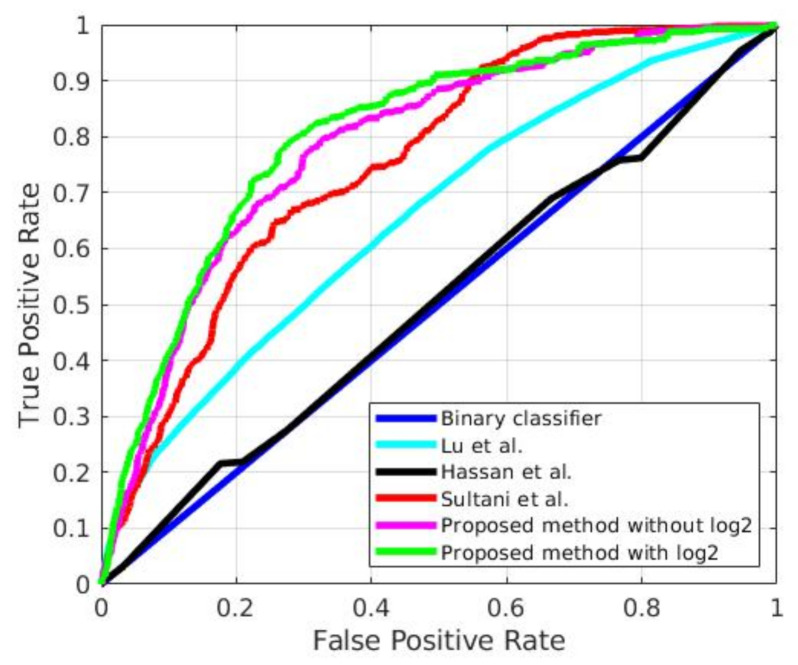
ROC comparison of the base binary classifier (blue), with the proposal in [12] (cyan), the one in [15] (black), the one described in [28] (red) and the proposed in this paper (pink and red, according to the different loss function.) in the UCF-Crime dataset.

The experiments have been conducted with the entire set of test videos of UCF-Crime with an average computational time of 1 min and 45 s for each video (with an average length of 2221 frames), what allows the proposal working at over 20 fps. Besides, the proposed methodology achieves in this condition, an AUC of 80.36% whereas the existing state-of-art methods achieve a maximum AUC of 78.66% justifying the outstanding performance of the proposal. It can also be observed in Figure 3, which displays the ROC comparison of the proposed approach (in red), as well as that of earlier methods.

**Figure 4:**
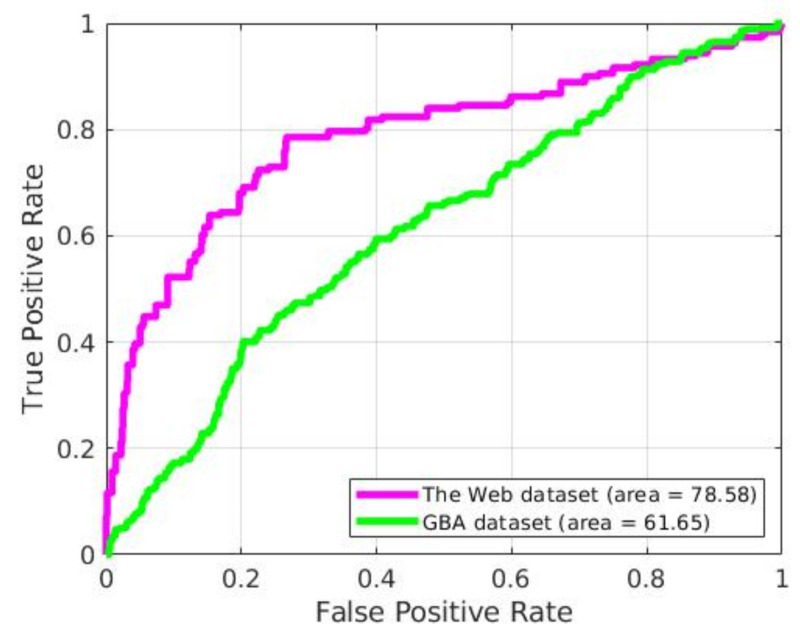
ROC of the proposal for GBA and The Web datasets.

The generalization capability of the proposed model validated using Figure 4 ROC curve obtained from implementing the method on GBA and Web datasets without fine-tuning. In our experiment with The Web and GBA datasets, we resized all the test videos into 320×240 dimensions (.mp4, H.264 codec) and manually annotated abnormal videos. The ROC in Figure 4 shows that our proposal for The Web dataset achieves an AUC of 78.58% which outperformed state-of-the-art, which was an AUC of 73% [37] method and for GBA dataset we have obtained 61.65%.

Results of the proposal on The Web dataset are better than those obtained within GBA one because some anomalous scenes in The Web dataset are similar to some in the UCF-Crime one. It means that the proposal already has some knowledge of Web anomalies before inserting any of its videos into the model. On the other hand, GBA data has no scene similar to those appearing in UCF-Crime, thus with a result less predictable.

It is worth highlighting that, although the scenarios in GBA dataset are totally different from those in UCF-Crime, the proposal is able to obtain valid results in anomaly detection without fine-tuning, thus ensuring its generalization capability.

To complete the quantitative analysis, Table 4 shows the precision, recall and f1-score for the three used datasets. Furthermore, there are also included these values for the baseline method [28] in the UCF-Crime dataset. Our introduced method achieved competitive results with a precision of 0.95, recall 0.91 and f1-score of 0.93 for abnormal class, whereas [28] method got precision of 0.94, recall 0.88 and f1-score of 0.91 for abnormal behavior. The presented results show that the proposal outperforms the baseline. It is noteworthy that these results have been obtained training only with UCF-crime, without fine-tuning for GBA and The Web datasets, so it allows demonstrating the generalization capability of the proposed method, that is able to detect anomalies even in different scenarios and with different characteristics. The proposed model attained the precision of 0.95, recall 1.00 and f1-score of 0.97 for abnormal activity on GBA dataset and the precision of 0.96, recall 0.98 and f1-score of 0.97 in the detection of abnormal activity on The Web dataset.

As it has been explained in the introduction, recognition of anomalous events or dangerous situation in real world applications in difficult, since the occurrence of abnormal events is minimal when compared to normal ones, and those anomalous events are complex, miscellaneous, and unexpected. Furthermore, they can change depending on the scenario. However, since the proposed method does not rely upon any specific kind of abnormal events, it can be applied in several different contexts, such as streets, airports, banks, shopping malls, etc., helping human operators to detect anomalous or dangerous situations (including crime detection, traffic analysis, etc.).

Regarding the computational cost, for real applications, it is desirable that the proposal works in real-time. The proposed work is able to detect anomalies with a speed over 20 fps, that can be enough for some applications, such as helping a security guard to know if there is an anomaly in the area under surveillance or detect a traffic accident. The use of more powerful hardware elements (GPU) can increase the speed, making it possible to use it in numerous real-world applications.

Having reported the quantitative results, qualitative ones are presented in Figure 2. In order to visualize the improved performance of the proposed method, both UCF-Crime dataset and real scenarios unseen by the algorithm from GBA are considered. The scenarios comprise both far-view and near-view video sequences.

The proposed methodology and the baseline method proposed by [28] are executed on all of them, and the anomaly responses from both the models are compared. The anomaly ground truth of each video is then plotted along with the anomaly score obtained from each proposal. The anomaly ground truth indicates a maximum anomaly score of 1, while represents normal with a minimum of 0.

For each result, they are shown, in the corresponding figure, four images from each selected sequence at the top, and the anomaly scores given by the two different architectures at the bottom, within the ground truth (in dotted green): Sultani’s one at the left side of the figure, and our proposal at the right side.

The first test (shown in Figure 2) is performed over the GBA dataset, corresponding to a far-view sequence in which a person falls. It comprises four scenarios namely: (a) a person walking, (b) the person falling down, (c) the person still lying on the ground, and (d) the person getting up. According to the ground truth, scenarios (b), (c), and (d) are considered anomalous, while scenario (d) is normal.

Within this context, as it can be seen in Figure 2, the proposed methodology yields high anomaly scores for the scenarios presenting the person’s fall ((b) and (c)) and getting up (d), whereas a low anomaly score for the normal event (person walking) in scenario (a). Besides, the model from Sultani’s paper gives a moderate score for scenario (b) and a low anomaly score for all remaining scenarios (a,c,d), which supposes a false negative. Thus, comparing the anomaly scores, the values assigned to scenario (b) by the proposed model are significantly high (near the maximum), so it can be concluded as an anomalous scenario. In the case of scenarios (c) and (d), the proposed model assigns a moderately high score while the one proposed by Sultani assigns a lower value.

**Figure 5:**
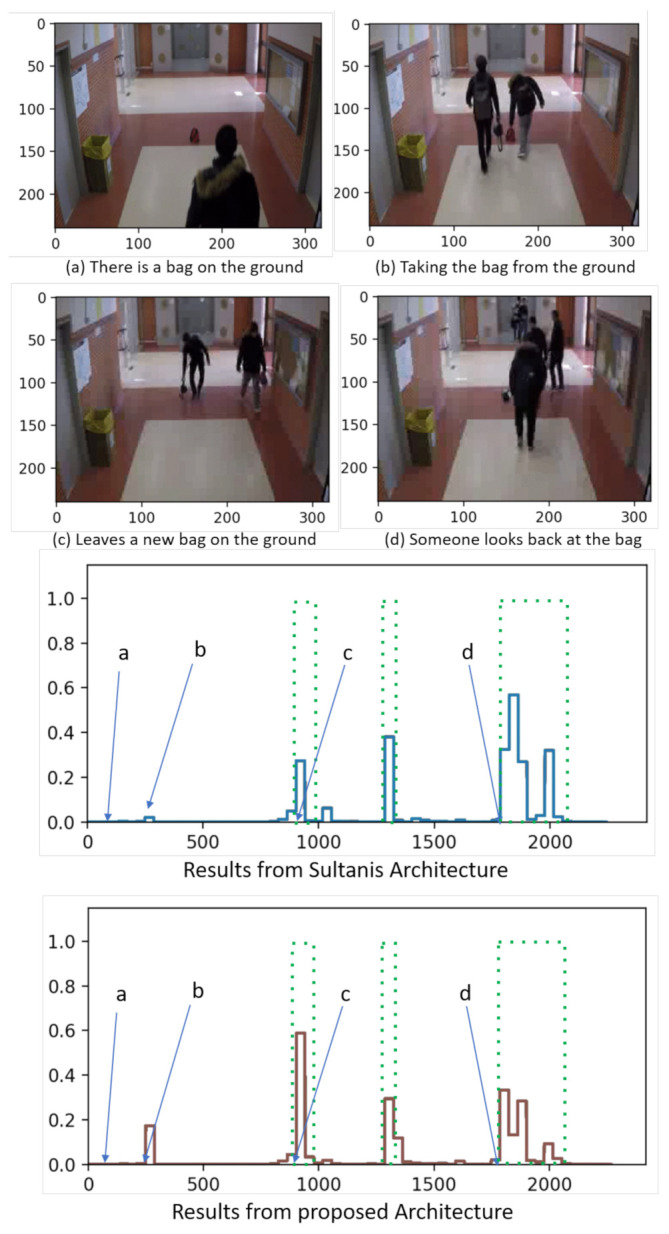
Qualitative visual results and comparison in scenes from GBA.

Subsequently, both models have been utilized to process a scenario from GBA in near-view, showing the obtained results in Figure 5. This second example is related to abandoning objects at standard smart spaces (like airports) that may be an important anomaly in video surveillance. It also comprises 4 scenarios: initially, in scenario (a) there is a bag on the ground. Scenario (b) depicts a person taking the bag from the ground, and in scenario (c) another person leaves a new bag on the ground. Finally, in scenario (d) someone looks back at the bag on the ground. According to ground truth, scenario (a) is considered normal, while the remaining ones are anomalous.

In this case, both the models have yielded high anomaly scores to the scenarios (c) and (d) and, hence, work similarly. Oppositely, just the proposal presented in this work detects the action of taking a bag, thus behaving better than Sultani’s one.

Thus, both in the case of far and near views of the GBA dataset, the proposed methodology achieves better performance than the proposed by Sultani.

Finally, they are graphically shown the results in two samples from the UCF-Crime dataset to compare again in them the results given by both methods.

**Figure 6:**
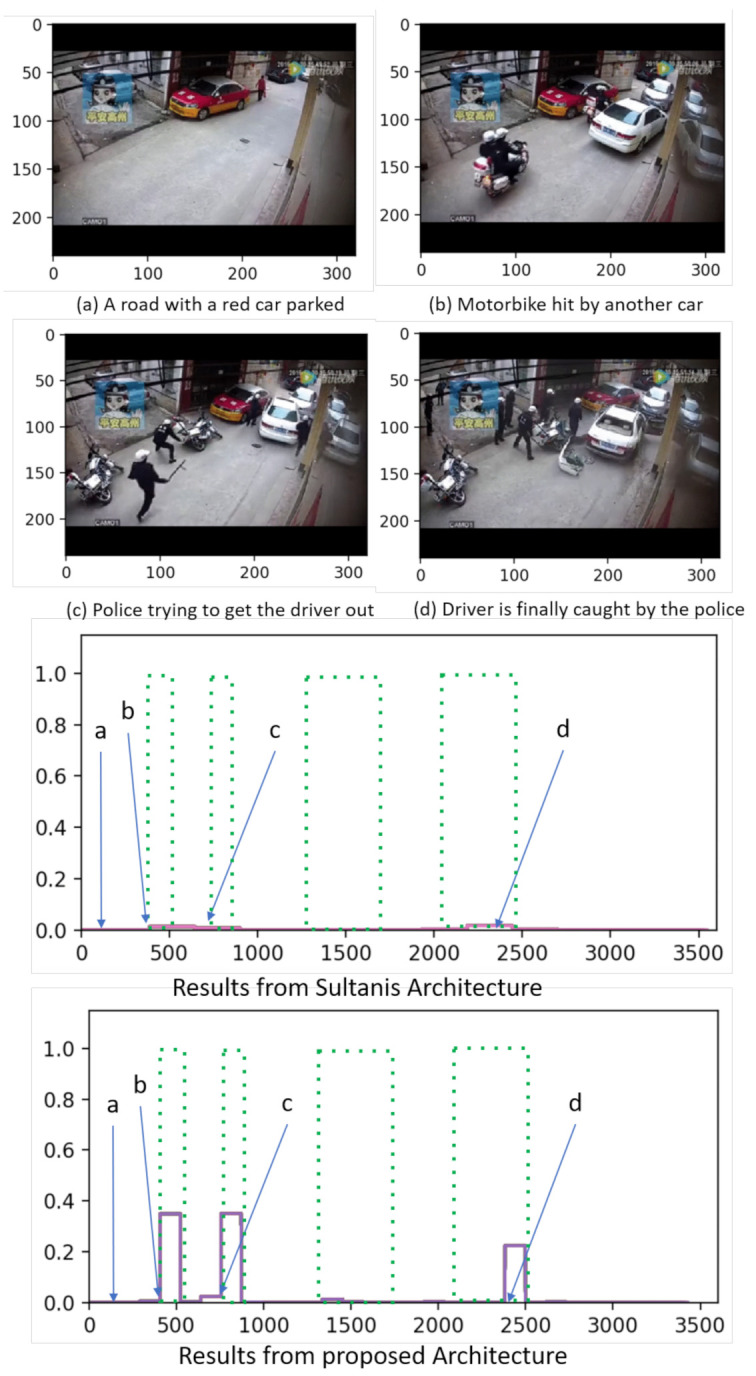
Qualitative visual results and comparison in scenes from UCF-Crime.

The first test is performed in an arrest situation, as shown in Figure 6. In this example, scenario (a) shows a road with a red car parked and people walking. Scenario (b) shows a motorbike being hit by another car. Scenario (c) shows the police trying to get the driver out of the hitting car, and scenario (d) displays the moment in which this driver is finally caught by the police. According to the ground truth, scenario (a) is a normal event while scenarios (b,c,d) are anomalous.

Comparing the results given by the proposed model with those generated by Sultani’s one in this example, our model yields higher anomaly scores for all anomalous scenarios, hence concluding that it performs better. Besides, both models detect the normal scenario in the same manner.

**Figure 7:**
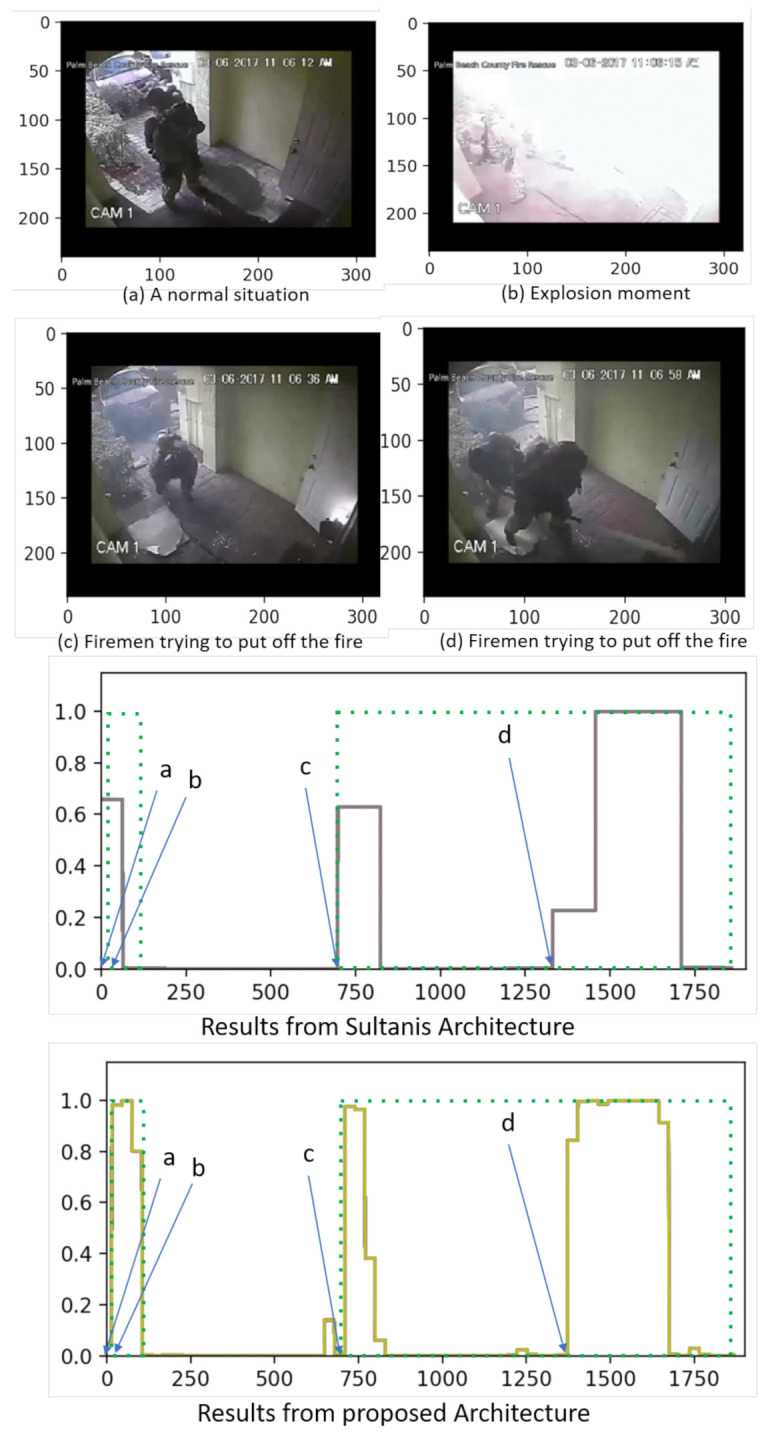
Qualitative visual results and comparison in scenes from UCF-Crime.

The second sample from UCF-Crime, in Figure 7, is an explosion. In this example, scenario (a) depicts a normal situation. Scenario (b) shows the explosion moment, and scenarios (c) and (d) present firemen trying to put off the fire. In the ground truth, scenario (a) is labeled as normal while scenarios (b,c,d) are labeled as anomalous.

In this comparison, our proposed method yields higher anomalous scores for scenarios (b) and (c) when compared to those provided by Sultani’s model. In the case of scenario (d), both models perform similarly. Thus, this example also projects the outstanding performance of the proposed model.

## 5. Conclusions and Future Works

Anomaly detection is explored in videos captured from surveillance cameras in the wild in real daily living scenarios. In the proposal, scene features are generated through a temporal convolutional 3D network (T-C3D). This extractor is pre-trained with the large-scale Kinetic dataset. The resulting features are input into a multiple instance learning (MIL) classifier, thus building a normality-anomaly model with an also new proposed ranking loss function, that aids in reducing the classification miss rate greatly.

The proposed approach achieves an AUC of 80.36%, outperforming all previous works of the related state-of-the-art and more precisely it achieves 1%, 3%, and 2% higher in precision, recall and f1-score for anomaly detection when comparing with the baseline model [28]. It is found to be more sensitive in the detection of anomalous videos, both within the UCF-Crime dataset, and the standard indoor realistic scenario of GBA.

Recognition of different anomalous activities in the real world is complicated. Since our proposed method does not rely upon any specific kind of normal and abnormal activity so it can use in various application scenarios such as streets, airports, banks, shopping malls, school or college campuses, etc., helping human operators to detect anomalous or dangerous situations.

However, the proposed approach faces challenges in handling videos and frames with fast motions, high density of people groups, low resolution, and poor illumination. The efficiency and robustness of the anomaly detection model in handling challenging situations can be improved in future works by taking motion information also into the account. In this context, a two-channel approach exploiting both RGB and optical flow information can be adopted, as in some proposals from the scientific community.

An important work-in-progress improvement is to modify the proposal to be able to run in test it in real-time, in order to obtain a stand-alone surveillance product. In fact, a real-time application for anomaly detection in the video-surveillance context needs high-speed detection in order to respond quickly and efficiently to crime and anomalous incidents. Currently, with our proposed model, we can detect normal and abnormal activity with a speed over 20 fps. We strongly believe that in our future work (using a two-channel approach), our model might be able to detect anomalies in real-time. For the pursuit demonstrator, the model should be tailored to detect anomalies within a time window of few seconds, tackling the problem as a time-labeling task. A reliability analysis of the proposed architecture results with training-testing ratios within a new dataset like 80%–20%, 60%–40% and 50%–50% is also under process, in order to obtain cost-efficient conclusions of the proposal. Some approaches towards this last approach are a near-future work of the one here presented.

## Figures and Tables

**Table 1:** Number of videos for training and testing, including normal and abnormal ones in UCF-Crime dataset.

Stage	# Videos	Normal	Abnormal
Training	1610	800	810
Testing	290	150	140

#: number.

**Table 2:** Number of sequences and individuals performing any of the 4 actions in GBA dataset [36].

Action	People	Sequences
Walk	13	72
Run	12	75
Sit down	16	78
Fall down	14	70

**Table 3:** Performance comparison using UCF-Crime dataset. The performance of the proposed in this paper appears in italics.

Model	Learning Algorithm	AUC
Binary classifier	Binary SVM classifier	49.99
Hassan et al. [15]	Unsupervised	50.66
Lu et al. [12]	Unsupervised	65.51
Sultani et al. [28]	Weakly Supervised	75.41
Zhang et al. [29]	Weakly Supervised	78.66
***Proposed method without*** $\log_{2}$	Weakly Supervised	***78.63***
***Proposed method with*** $\log_{2}$	Weakly Supervised	***80.36***

**Table 4:** Precision, recall and f1-score obtained with the proposal for anomaly detection in UCF-crime, GBA and The Web datasets, and comparison with [28] in UCF-Crime.

Dataset	Model	Precision	Recall	F1-Score
GBA dataset	Proposed method	0.95	1.00	0.97
The Web dataset		0.96	0.98	0.97
UCF-Crime		0.95	0.91	0.93
	Sultani et al. [28]	0.94	0.88	0.91
